# The Corset

**Published:** 1890-12

**Authors:** 


### THE CORSET.

Extract of a paper entitled " Artistic Dress," read before the Chicago " Society for the Promotion of Physical Culture and Correct Dress," by Frances M. Steele :

If we reverence the Creator, no human form can be beautiful to us that is not natural and healthy. A small waist is only pretty when harmonizing with general slightness. The dainty waist of the poets is precisely that flexibility which is a natural characteristic of youth. It is later replaced by a beauty of greater dignity. That flexibility is one quality destroyed by corsets. When the shoulders spread out above and the hips poke out below, a small waist is only a deformity. It is only because modern men and women have been accustomed to such a departure from nature that the deformity is admired, or even tolerated. The curves of the natural body are all outward curves, one gently rising out of the other ; but the chief curves of a corset are inward curves, which are utterly incorrect—exactly opposed to those elements which Ruskin has taught us are beautiful in a curve. Fixed angles are monstrous except where nature has placed them, at the juncture of the limbs with the trunk.

Another ugly feature of the corset is the whalebones, whose rigidity obscures that rippling movement of the body which is one of its chief beauties. So far as that quality which artists speak of as the " sentiment of the muscles " is concerned, the woman might as well be incased in cast iron, or be one of those " stylish " squaws who persistently offer you a bundle of cigars at the door of a tobacco shop. Other ugly features are the hard cross line of the bust, which distinctly shows, and the whalebones and lacing can generally be seen through the dress behind.

The ampler the form the less can good taste consent to its compression. Every one knows that a large person looks less large when moving with ease and grace. Tight dressing destroys both. The sudden bulges and violent amplitudes it displays are distressing to the sense of modesty. Ineffective, imperfect struggles to move about are distressing to the sense of beauty and grace. These things are positively ugly. And all this unlovely spectacle for what ? Pleasure ? Certainly not. Beauty ? Certainly not. For nothing but the gratification of a depraved taste. For just the reason, and no other, that the savage sticks a bone through her lip to make it hang down below her chin. Nature is grievously insulted, and often avenges herself. The epitaph—

> Here lies a girl whose brief, brief days,
> Were briefer still for wearing stays, if often repeated, would tell more truth than is commonly found in grave-yards.

